# Inflammation, infection, and allergy of upper airways: new insights from national and real-world studies

**DOI:** 10.1186/s13052-020-0782-z

**Published:** 2020-02-10

**Authors:** Attilio Varricchio, Ignazio La Mantia, Francesco Paolo Brunese, Giorgio Ciprandi

**Affiliations:** 1 UOSD Video-Endoscopia delle VAS, P.O. San Gennaro - ASL Napoli 1-centro, Naples, Italy; 2 ENT Department, University of Catania, Catania, Italy; 3 Primary Care Paediatrics, ASL Caserta, Caserta, Italy; 4 Allergy Clinic, Casa di Cura Villa Montallegro, Via Boselli 5, 16146 Genoa, Italy

**Keywords:** Upper airways, Allergy, Inflammation, Infection, Anatomy, Physiology, Therapy

## Abstract

The upper airways (UA) should be considered as a functional unit. Current functional anatomy divides URT in three, mutually dependent, “junction boxes”: i) the ostio-meatal complex (OMC), ii) the spheno-ethmoidal recess (SER), and iii) the rhinopharynx (RP). Correct ventilation and effective mucociliary clearance of these sites significantly affect the healthy physiology of the entire respiratory system. The OMC, SER, and RP obstruction is the first pathogenic step in the inflammatory/infectious cascade of UA disorders. The inflammation of the respiratory mucosa is the main pathogenic factor for airway obstruction. Moreover, bacterial biofilm (a strategy modality of bacterial survival) is an important local cause of systemic antibiotic ineffectiveness, recurrent infections, and antibiotic resistance. Health microbiota guarantees UA wellness; on the contrary, dysbiosis promotes and worsens UA infections. Allergy, namely type 2 inflammation, is a common cause of UA obstruction such as promoting in turn infections. Fiberoptic endoscopy is a mandatory diagnostic tool in clinical practice. Nasal cytology, mainly concerning flow cytometry, allows defining rhinitis phenotypes so allowing a precision medicine approach. Several conventional therapeutic approaches are available, but efficacy and safety should be ever properly considered before the prescription. Also, complementary medicine plays a fruitful role in the management of UA diseases. National and real-world studies are reported and discussed as they may be useful in daily clinical practice.

## Background

Inflammation, infection, and allergy of upper airways constitute relevant pathophysiologic mechanisms involved in the most frequent diseases in childhood. The current Commentary summarizes the pathogenic pathways of upper airways diseases (UAD) sharing these mechanisms and presents and discusses the outcomes accomplished by national studies conducted in real-world settings. The choice of selecting this kind of studies was grounded in two pragmatic assumptions. Clinical features of UAD significantly vary depending on the geographic area, therefore, findings from foreign countries cannot be simply transmuted and generalized to the local reality [1, 2]. More and more attention had paid to the real-world studies as they may provide information more adherent to the daily practice as recruit patient populations that reflect the actual situation faced by any paediatrician [3, 4].

### Functional anatomy and pathophysiology

The airways should be globally considered a functional unit as there is strong evidence that upper airways (UA) and lower airways (LA) are closely linked [5, 6]. UA and LA share common epidemiologic, anatomic, physiological, and pathophysiologic mechanisms. A paradigmatic example is a rhino-bronchial syndrome characterized by a spread of inflammatory and infectious events from the nose to the bronchi [7, 8].

In the rhino-sinus-pharyngeal district, there are three important pathophysiologic sites: i) the ostio-meatal complex (OMC), ii) the spheno-ethmoidal recess (SER), and iii) the rhino-pharynx (RP) as previously pointed out [9, 10]. The OMC, located on the lateral wall of the nose and bordered by the low and middle turbinate, is the space where the anterior rhino-sinus system, including the frontal sinus, the maxillary sinus, and the anterior ethmoidal sinus, is devoted to draining the secretions.

The SER, located in the lateral wall of the nose, behind the OMC, bordered by the middle and superior turbinate, drains the posterior rhino-sinus system, including the posterior ethmoid sinus and the sphenoid sinus. The RP is the site where the post-nasal drip (such as posterior rhinorrhea) starts, it may cause descending pharyngo-tracheobronchitis. In the RP, the adenoids are located too. RP inflammation/infection is also implicated in the pathogenesis of otitis media. Moreover, RP is the site of the so-called “microbial bank” and the preferred place of bacterial biofilm [6].

Correct ventilation and effective mucociliary clearance of these three pathophysiologic junction boxes guarantee the healthy physiology of the whole respiratory system. In these sites, it occurs the filter of the inhaled air, the enrichment with nitric oxide, which in turn regulates the ciliary motility, may prevent viral replication and regulates the bronchial muscular tone [11, 12]. Therefore, the OMC, SER, and RP obstruction is the first essential pathogenic step in the cascade of inflammatory events of the rhino-sinus-pharyngeal district, often complicated by infections.

The infections may involve OMC and/or SER, causing an anterior and/or posterior rhinosinusitis [10]. Also, obstruction of OMC and/or SER causes an inflammatory reaction and posterior rhinorrhea, involving the microbial bank of the nose and pharynx, that may spread to the tubaric-tympanic-mastoid unit or lower airways. As a consequence, the rhino-bronchial syndrome occurs. Alternatively, it may involve the tympanic cavity, through the Eustachian tube, causing otitis media [13]. Also, in the case of postnasal drip, secretions (containing microbes and pro-inflammatory substances) may spread to middle-lower airways [14]. The inflammation of the respiratory mucosa is the main pathogenic factor inducing airways obstruction [15].

Infectious diseases mostly affect younger children, due to their immunological immaturity. On the contrary, inflammatory diseases and allergy mostly affect older children, adolescents, and adults [10, 14]. Acute infectious diseases have essentially a viral origin, while recurrent and chronic forms have mostly a bacterial origin. The last is due to a lack of recovery of acute infection, sustained by local (adenoids hypertrophy, anatomic, and functional malformations) and/or systemic causes (allergy, mucociliary alterations, immunological defect) [14, 15]. The biofilm is an important local cause of recurrent diseases: biofilm is a strategic modality of survival set up by bacteria and the main cause of their resistance to systemic antibiotic therapy [16]. A considerable presence of biofilm has been demonstrated in the RP of children suffering from recurrent infections of upper airways [17]. Biofilm can release, time after time, numerous bacterial colonies that cause the recurrence of respiratory infections. Both infectious and inflammatory diseases share the same pathophysiological mechanisms: the respiratory mucosa shows a wide submucosal diffusion of immune-inflammatory cells which amplify inflammation [15, 18]. In this context, allergic inflammation may promote a vicious circle that allows the predisposition to infection recurrence and increase symptom severity.

### Recurrent respiratory infections

Children with recurrent respiratory infections (RRI) represent a compelling challenge for the otolaryngologist and the paediatrician in daily practice. It was reported that at least 6% of Italian children younger than 6 years old had RRI [19]. The definition of RRI is still debated and complex. The RRI definition, proposed by an Italian panel of experts, considered a series of exclusion criteria (including absence of primary or secondary immunodeficiency, cystic fibrosis, primary ciliary dyskinesia, and airways malformations) and the presence of at least one of the following conditions: i) > 6 annual RI; ii) one or more monthly RI from October to February; iii) > 3 RI involving lower airways [20]. Moreover, different definitions have been proposed for each airway disorder: 1) for recurrent pharyngotonsillitis > 7 episodes of pharyngotonsillitis in 1 year, > 5 episodes/year for two consecutive years or > 3 episodes/year for three consecutive years [21]; 2) for recurrent otitis media > 3 episodes in 6 months or > 4 episodes/year [22]; 3) for recurrent lower RI > 3 episodes of bronchitis, bronchiolitis, or pneumonia [23]. However, differentiating lower and upper respiratory tract infections may be difficult, because symptoms and signs overlap and both may be present at the same time [24]. However, the duration and severity of RI should be also evaluated as reported in recent studies. A systematic review, concerning 5427 children, reported that common cold resolved by 15 days [25]. A Finnish study reported that the 90th percentile of the number of days in the year with respiratory disease was 98; therefore, RRI was diagnosed in children (younger than 2 years) who exceed this cut-off; the median was 9.6 RI/year [26].

Based on these issues, it is evident that RRI has a significant impact on pharmaco-economy and cause a relevant burden for both the family and society as they have frequently physician office and emergency room visits, often use antibiotics or are hospitalized for severe and complicated RI.

It has been postulated that many risk factors can account for promoting and/or causing RRI, including prematurity, preschool age (for a relative immaturity of the immune system), early attending at nursery school, air pollution, home dampness, passive exposure to tobacco or vape fumes, low socioeconomic level, overcrowding, and allergy [27]. Moreover, it has been hypothesized that the allergic diseases may play a particular role in promoting the RI recurrence as the physiological immune response is impaired in allergic subjects and allergic inflammation favours predisposition to infections. Type 2 immune response is defined by increased secretion of prototypical cytokines IL-4, IL-5, and IL-13 that promote, maintain, and amplify type 2 inflammation, characteristic of allergic patients [28]. However, there are different endotypes of type 2 immune response, namely, type 2 high and low, thus an adequate assessment of the immunopathological profile should be addressed in allergic patients [29]. As a consequence, subjects with allergic disorders may have a functional defect of type 1 immune response that is relevant in fighting infections. It has been reported that patients suffering from allergic diseases are prone to have more numerous and severe infections than non-allergic subjects [30, 31]. On the other hand, this situation could be reverted by allergen-specific immunotherapy [32]. Also, viral infections may increase the probability of contracting frequent RI because of the high number of circulating viruses and numerous subtypes [33]. Viral infections are predominant, but bacterial super-infections may frequently appear. Consequently, there is an overuse/misuse of antibiotics by primary care doctors that in turn induces antibiotic resistance [34, 35]. Moreover, biofilm causes frequent antibiotic unsuccess and 25–45% of children with severe RRI need surgical intervention [36, 37]. On the other hand, there is no available biomarker able to identify children at risk of RRI at present.

### Recurrent acute otitis media

Acute otitis media (AOM) is an ear disease defined by signs or symptoms of acute infection [38]. AOM is the most common bacterial infection in children [39–43]. Consequently, AOM is the most common reason for antibiotic prescription in the pediatric age [44, 45]. Almost all children experience at least one episode of AOM during childhood. Therefore, the burden of AOM is relevant both concerning the direct (healthcare expense) and indirect costs (loss of school attendance and parental workdays) and negatively impacts on quality of life of children and their parents. Moreover, antibiotic overprescription is the main cause of the increase of multidrug-resistant microbes as well as for the occurrence of adverse reactions [46, 47]. For these reasons, several guidelines on AOM management were performed to optimize management and therapy [44, 45].

Notably, some children with AOM tend to be otitis-prone, such as to have a frequent recurrence of AOM (RAOM). So, the identification of factors involved in the recurrence may have a practical interest. RAOM represents, therefore, an intriguing challenge in the clinical practice for both the paediatrician and the otorhinolaryngology (ORL) specialist.

The AOM has infectious pathogenesis and is characterized by the trio: hypoacusis, fever, and otodinia. The AOM diagnosis requires adequate procedure and precise differential diagnosis, mainly concerning OME that has always inflammatory pathogenesis and clinical presentation is characterized by hypoacusis, never associated with fever and/or otodinia. A correct otoscopy is mandatory to differentiate viral AOM (the eardrum is flat and the bright triangle is visible) from bacterial AOM (eardrum is bulging and bright triangle is not visible). Moreover, AOM antibiotic therapy is controversial as many guidelines suggest watchful waiting for mild-moderate episodes in children > 2 years aged. Moreover, the prevention of RAOM is overwhelmingly desirable, even though it is still debated. At present, there is no convincing evidence of preventing RAOM by the proposed treatments both conventional and not [44–47]. In this regard, the failed recovery from rhino-pharyngeal disease (as the microbial bank is not removed) may promote the AOM recurrence. To solve RAOM, topical treatments and probiotics have been proposed [44]. Therefore, as there is no convincing preventing and effective preventive treatment for RAOM, to know predictive factors for RAOM could be fruitful from a pragmatic point of view. Anyway, as very recently reported, the pragmatic approach in primary care is an overuse of broad-spectrum antibiotics [48].

### Adenoiditis

Adenoids belong to the Waldeyer’s ring; they are exposed to microbes, allergens, and environmental irritant factors [49]. Adenoids play a crucial role in promoting and maintaining a correct innate and adaptive immune response [50, 51]. The adenoids size trend to growth during childhood, with a maximum between 2 and 5 years; after 10 years of life, they progressively reduce their volume. Recurrent respiratory infections are the most important cause of adenoidal hypertrophy (AH). AH has clinical relevance as large adenoids are an important reservoir for bacteria. Also, resident bacteria tend to persistently remain into adenoids by producing biofilm [52]. So, they can become resistant to defensive mechanisms and antibiotics. Moreover, adenoiditis is clinically relevant as it may spread to other organs, worsening otitis, rhinosinusitis, and lower airways infections [53]. Therefore, adenoiditis represents a relevant burden in children, mainly when recurrent. Preventing recurrent adenoiditis, often associated with AH, represents a compelling challenge in clinical practice. Acute adenoiditis is defined by signs and symptoms of acute infection, including nasal obstruction, posterior mucopurulent discharge, oral breathing, dry mouth, closed posterior rhinolalia, halitosis, and fever. Recurrent adenoiditis associated with AH causes sleep disorders, impaired craniofacial growth, reduced attention, and also enuresis. Consequently, recurrent adenoiditis is a common reason for antibiotic prescription in the paediatric age. A preventive strategy could be therefore very fruitful. In this regard, an interesting option has been highlighted by the study of the microbiome [54, 55]. On the other hand, the physiological rhino-pharyngeal microbiome does constitutively inhibit the growth of local pathogens. It has been demonstrated that the administration of “good” bacteria (the so-called “Bacteriotherapy”) could exert preventive effects on infections. In this regard, Bacteriotherapy was successfully administered as a nasal spray for preventing recurrent acute otitis media [56, 57].

### Rhinosinusitis

Sinusitis usually refers to inflammation of the nasal sinuses. However, as it is very commonly associated with the inflammation of the nasal mucosae, such as rhinitis, the term rhinosinusitis (RS) has been considered more correct [58]. In clinical practice, acute RS (ARS) is diagnosed in the presence of nasal symptoms, including nasal congestion and rhinorrhea, persisting for more than 7–10 days without any improvement [59, 60]. RS differs from the common cold because this is usually self-limiting and usually resolves by 7–10 days [60–62]. The symptoms of ARS tend to resolve within 3–4 weeks; however, if sinus inflammation persists (regardless of the medical management), it is evolving to chronic RS (CRS), defined by a duration longer than 8–12 weeks [63]. Therefore, the diagnosis of RS often relies on the clinical ground, including the duration of nasal symptoms, the characteristics of nasal discharge (purulent), and other symptoms, such as facial pain and fever. However, fibre-optic endoscopy is the gold standard diagnostic tool and its use is mandatory in clinical practice [64]. Computerized tomography (CT) may be required whenever the suspicion of extra-sinus complications should arise [65, 66]. Moreover, CT is useful to detect nasal polyps in CRS patients. According to the endoscopic and/or radiological findings, there are two main CRS phenotypes: CRS with nasal polyposis (CRSwNP) and CRS without nasal polyposis (CRSsNP).

### Common cold

The common cold is surely the most common infective disease at any age. Viruses are the main cause of the common cold, mainly concerning rhinovirus. Usually, the diagnosis is clinically-grounded. It is a self-limiting infection but could worsen and cause acute rhinosinusitis, mainly in presence of local predisposing factors, including adenoid hypertrophy, nasal polyposis, septal deviation, promoting mechanical obstruction or systemic factors, including allergy, ciliary dyskinesia, and immune deficiency. Acute rhinosinusitis could be suspected when an episode of common cold lasts for more than 10 days or symptoms worsen after 5 years [67]. Specific treatments of the common cold do not exist as well as vaccination is ineffective. Therefore, symptomatic treatment is usually prescribed in common practice. However, early treatment addressed to remove secretions and dampen microbial load could be desirable.

### Recurrent rhinosinusitis

Recurrent rhinosinusitis (RRS) is defined by multiple episodes of acute rhinosinusitis (ARS), mainly of bacterial aetiology [51]. As defined by International guidelines [63, 68], the diagnosis of ARS is based on the clinical history and endoscopic assessment. As punctually pointed out by the guidelines, treatment includes topical anti-inflammatory drugs and antibiotics use based on clinical ground. On the other hand, anti-inflammatory agents may have relevant side effects, mainly in children. Also, antibiotic overuse is frequently associated with outgrowth of multi-resistant microbes as above mentioned. An effective RRS prevention might significantly affect the risk of complications, medical costs, and social and family impact. On the other hand, many past attempts of prevention were usually expensive, long-lasting, and seldom fruitless or accompanied by adverse events. Therefore, preventing RRS using an alternative way might represent an interesting and stimulating issue as recently reported [69]. RRS prevention may include bacteriotherapy, probiotic administration, vitamins, oligo-elements, or the use of immune-stimulant compounds [70].

### Allergic rhinitis

Allergic rhinitis (AR) is the most common immune-mediated disorder as it may affect up to 40% of the paediatric population [71]. AR has clinical relevance as is frequently associated with comorbidities, including other allergies, asthma, rhinosinusitis, recurrent respiratory infections, otitis, adenoid hypertrophy (AH) and tonsillar hypertrophy (TH), as recently reported by several recent studies [72–75]. Moreover, the possible correlation between AR and AH-TH has been investigated by some studies which reported a positive association between the two disorders [76–80]. Atopy, such as the genetic predisposition to allergic diseases, is also common in AR children.

It is well known that the rhinologist visits children complaining of nasal symptoms daily. A pale mucosa in the nasal cavity has been traditionally considered a sign suggesting allergic rhinitis by most ORL specialists [81, 82]. However, it has been evidenced that turbinate hypertrophy is a sign with higher predictive reliability to suspect allergic rhinitis during an ORL visit both in children and adults [83, 84]. Nasal obstruction is, in fact, a common symptom of allergic rhinitis as it has been reported that it is present in about 80% of children with allergic rhinitis [85]. Consistently, it has been reported that also bronchial airflow limitation, documented by simple spirometry, may be able to suspect the presence of sensitization, such as positive skin prick test [86, 87]. Furthermore, it has to be highlighted that to define a diagnostic marker there is the need to fulfil a series of pragmatic requirements as recently pointed out [88].

AR is characterized by typical nasal symptoms and IgE-mediated inflammation [89]. A type 2 inflammation is initiated by causal allergen exposure [90]. Mediators and cytokines are actors involved in the scenario of inflammatory events. A typical pattern of inflammatory cells infiltrates the nasal mucosa: it includes eosinophils, neutrophils, and mast cells. A clear-cut association between allergen exposure and nasal inflammation has been documented exhaustively using these criteria in the past [91–93]. AR treatment is based on antihistamines and intranasal corticosteroids administration [94]. These medications are usually effective and safe but exert symptomatic activity that shortly disappears after discontinuation. The only cure for AR is allergen-specific immunotherapy that consists of the administration of the causal allergen for a long period [94]. Ancillary treatments may include nasal irrigation, non-steroidal anti-inflammatory compounds, and preventive therapies as discussed later.

### Non-allergic rhinitis (NAR)

Non-allergic rhinitis is an umbrella definition that includes different phenotypes and endotypes. The most common types of non-allergic rhinitis are inflammatory rhinitis and vasomotor rhinitis [95]. Inflammatory rhinitis is characterized by nasal symptoms sustained by a non-specific hyperreactivity and nasal non-IgE mediated inflammation. The diagnosis is based on clinical history, negative allergic tests, and the detection of inflammatory cells infiltrating the nose. Different types of NAR have been identified and classified considering the predominant inflammatory cells: non-allergic rhinitis with eosinophils (NARES), non-allergic rhinitis with mast cells (NARMA), non-allergic rhinitis with eosinophils and mast cells (NARESMA), non-allergic rhinitis with neutrophils (NARNE).

The treatment of non-allergic inflammatory rhinitis is merely symptomatic, using topical or oral antihistamines, topical anticholinergic drugs, and intranasal corticosteroids. Nasal lavage may alleviate symptoms.

Vasomotor rhinitis is caused by nasal hyperreactivity mainly due to exaggerated neural reflex. The treatment may be advantaged also by topical anticholinergic agents [95].

### Pragmatic work-up

In clinical practice, AR diagnosis is based on the consistency between clinical history and the assessment of allergen-specific IgE [94]. Nasal allergic inflammation may be documented by nasal cytology as previously proposed [96], even though the main criticism is the lack of standardized criteria. In this regard, a series of statistical criteria should be required to standardize a test [88]. Very recently, a standardization of nasal cytology has been reported [97].

On the other hand, some studies investigated nasal inflammation using flow cytometry, even though most of them aimed at evaluating issues far from conventional rhinology [98–104]. Flow cytometry allows defining a series of additional aspects in comparison with traditional nasal cytology, including the cellular volume and density, the antigenic and genetic cellular pattern, and the functional state, such as activation. Moreover, flow cytometry is automated and well standardized, so it may be considered as a precise and accurate method to analyze the cellular pattern in nasal inflammation. In this regard, it has been recently reported that flow cytometry is an adjunctive and reliable tool in the work-up of AR [105]. Also, nasal cytology, including flow cytometry, is fundamental to diagnose inflammatory non-allergic rhinitis as it is the unique test able to identify and count inflammatory cells in patients with non-allergic rhinitis [106].

Another useful clinical parameter, that is very simple and easy, is the visual analogue scale (VAS). VAS is a psychometric test able to assess the perception of symptoms and is commonly used in clinical practice. VAS also well correlates with objective measurement of nasal obstruction and can be re-assessed to evaluate change over time [107, 108].

However, it has to be underlined that nasal fibreoptic endoscopy is the gold standard diagnostic tool in the management of all UA disorders. At present, endoscopy is a mandatory and unavoidable step in the ORL work-up as it gives unique information about the anatomy and morphology of UA [109].

### Therapeutic strategies for UA diseases based on local and real-world studies

The burden and cost of inflammatory, infectious, and allergic disorders involving UA are increasing worldwide. Thus, strategies need to change management to support the transformation of the healthcare system for integrated care as recently proposed [110]. In this regard, integrated care pathways are structured multidisciplinary care plans that detail the key step of patient care [111]. They promote the application of guideline recommendations to clinical practice [112, 113]. Therefore, a multidisciplinary approach should include both diagnostic procedures and therapeutic strategies.

There is evidence that several therapeutic options may be effective in the treatment of UA diseases, as demonstrated by several studies that investigated the effectiveness and safety of different medication classes. In this regard, a synthetic review of a series of studies exploring the innovative use of conventional medications or the clinical application of complementary medicine is presented. As considered in the introduction, we selected studies conducted in Italy and real-world setting as they could realistically reflect what happens daily in clinical practice. Moreover, most of them were performed following a randomized and controlled design.

Nasal irrigation with hypertonic saline significantly reduced the adenoid size in children with adenoid hypertrophy [114]. Salso-sulfide thermal water was successfully used in children with RRI [115]. Consistently, salso-bromo-iodine thermal water reduced postnasal drip-related cough in children suffering from acute upper airway infection [116]. Antibiotics, such as tobramycin and thiamphenicol, were intranasally administered using a nasal micronized douche in children with acute rhinopharyngitis: both treatments significantly reduced the symptom severity [117, 118]. An original therapeutic strategy was adopted in children with perennial allergic rhinitis, i.e. a continuous schedule; long-term cetirizine administration significantly improved symptoms and reduced the use of symptomatic drugs in comparison with on-demand cetirizine [119]. Another study demonstrated that fexofenadine, a second-generation antihistamine, was able to significantly reduce nasal congestion in patients with perennial allergic rhinitis [120]. Corticosteroids, the most potent anti-inflammatory drug, may also reduce adenoid volume if administered topically in children with adenoid hypertrophy, as reported for intranasal flunisolide [121]. Notably, it was also demonstrated that nasal flunisolide application could also reduce the adenoid surgery [122]. Also, intranasal flunisolide, a topical corticosteroid, significantly affected nasal symptoms in patients with non-allergic rhinitis [123]. Flunisolide is also indicated in chronic rhinosinusitis as it is effective and has an optimal quality/cost ratio [124]. However, the nasal device and the excipients are relevant factors for improving the prolonged efficacy, safety, preference, and compliance as demonstrated for mometasone furoate nasal spray [125]. In particular, mometasone has a favourable safety profile also in paediatric patients as recently reported [126].

Considering complementary medicine, there is growing evidence demonstrating its useful role in the management of upper airway diseases. In this regard, hyaluronic acid exerts important anti-inflammatory activity associated with immune-modulatory and lubricant effects in children with bacterial acute rhinopharyngitis [127], in patients with allergic, non-allergic, and mixed rhinitis [128], and patients after functional endoscopic sinus surgery [129]. Resveratrol, a phenol characterized by anti-viral activity and anti-inflammatory activity, may significantly reduce nasal symptoms in children with pollen-induced allergic rhinitis [130]. Also, resveratrol plus carboxymethyl-β-glucan reduced the number of respiratory infections in children with allergic rhinitis [131]. This result was consistent with a similar study conducted in children with RRI [132]. An empiric approach in children with secondary sinonasal headache, consisting of nasal aerosol containing pirometaxine and copper sulfate associated with pidolate magnesium and exclusion diet, significantly improved symptom severity [133]. Cucurbitacins extract, as glycosylates triterpenes, resolved otitis media with effusion in 93.3% of treated children [134]. An oral nutraceutical, containing quercetin, extract of *Perilla*, and vitamin D3, was investigated in a randomized controlled study conducted in children with allergic rhinitis. The results provided evidence that this multicomponent nutraceutical significantly reduced the occurrence of clinical worsening and prevent rhinitis exacerbations in comparison with placebo [135, 136]. Probiotics are widely self-administered by the patients, but scientific evidence is scarce. *Lactobacillus reuteri* has been employed in asthmatic children: it significantly reduced bronchial inflammation and respiratory symptoms [137]. Moreover, probiotics may reduce the occurrence of infectious rhinitis which is commonly associated with allergic rhinitis [138]. If associated with vitamin D3, *L reuteri* provided more interesting outcomes [139]. Finally, as above discussed, bacteriotherapy, such as the administration of “good” bacteria (usually saprophytic), may be a promising approach in preventing respiratory infections as demonstrated in children with recurrent acute otitis media [140]. A preliminary study showed that bacteriotherapy could also prevent adenoid surgery [141]. More recently, it has been reported that also oral bacteriotherapy may significantly reduce the number of streptococcal infections, the use of antibiotics, and the scholar absences in children with recurrent streptococcal pharyngotonsillitis caused by Group A β-haemolytic *Streptococcus* [142].

Enoxolone, a potent anti-inflammatory, and immunomodulatory molecule, associated with mannitol, an osmotic anti-oedema agent, have been demonstrated able to improve the nasal mucociliary transport time [143], to reduce nasal eosinophilia in allergic children [144], and significantly reduce the severity of nasal congestion [145, 146].

### Future perspectives

There is a growing body of literature about a new promising approach to treat upper airway disorders using biologics [147]. Many trials have been conducted using biologics, such as omalizumab, a monoclonal antibody targeted toward IgE, mepolizumab, an anti-IL-5 agent, duplizumab, and anti-IL-4 and IL-3 biologic, to treat upper airway disorders, including allergic rhinitis and chronic rhinosinusitis with nasal polyps [148–150]. The outcomes were meaningful, however, it should be noted that the indication in upper airway diseases is still off-label.

## Conclusion

Upper airways should be adequately investigated performing an appropriate workup, including fibreoptic endoscopy and cytology. Inflammatory UA diseases should be treated with effective and safe medications. Respiratory infections deserve adequate observation, antibiotics should be prescribed with careful attention, and preventive strategy should be preferred. Allergic disorders can be successfully treated with specific therapy and allergic symptoms can be optimally relieved with medications. However, inflammation, infection, and allergy frequently coexist, therefore an integrated multidisciplinary approach should be considered in clinical practice. Also, conventional treatment may be opportunely associated and integrated using complementary medicine.

## Data Availability

Not applicable.
